# Gender differences in functional connectivities between insular subdivisions and selective pain-related brain structures

**DOI:** 10.1186/s10194-018-0849-z

**Published:** 2018-03-14

**Authors:** Yu-Jie Dai, Xin Zhang, Yang Yang, Hai-Yan Nan, Ying Yu, Qian Sun, Lin-Feng Yan, Bo Hu, Jin Zhang, Zi-Yu Qiu, Yi Gao, Guang-Bin Cui, Bi-Liang Chen, Wen Wang

**Affiliations:** 1 Department of Radiology & Functional and Molecular Imaging Key Lab of Shaanxi Province, Tangdu Hospital, the Military Medical University of PLA Airforce (Fourth Military Medical University), 569 Xinsi Road, Xi’an, Shaanxi Province 710038 China; 2 Department of Obstetrics and Gynecology, Xijing Hospital, the Military Medical University of PLA Airforce (Fourth Military Medical University), 15 West Changle Road, Xi’an, Shaanxi Province 710032 China; 3 Department of Clinical Nutrition, Xijing Hospital, the Military Medical University of PLA Airforce (Fourth Military Medical University), 15 West Changle Road, Xi’an, Shaanxi Province 710032 China; 4 Student Brigade, the Military Medical University of PLA Airforce (Fourth Military Medical University), 169 West Changle Road, Xi’an, Shaanxi Province 710032 China

**Keywords:** Gender differences, Insular subdivisions, Pain, Functional connectivity, Resting-state

## Abstract

**Background:**

The incidence of pain disorders in women is higher than in men, making gender differences in pain a research focus. The human insular cortex is an important brain hub structure for pain processing and is divided into several subdivisions, serving different functions in pain perception. Here we aimed to examine the gender differences of the functional connectivities (FCs) between the twelve insular subdivisions and selected pain-related brain structures in healthy adults.

**Methods:**

Twenty-six healthy males and 11 age-matched healthy females were recruited in this cross-sectional study. FCs between the 12 insular subdivisions (as 12 regions of interest (ROIs)) and the whole brain (ROI-whole brain level) or 64 selected pain-related brain regions (64 ROIs, ROI-ROI level) were measured between the males and females.

**Results:**

Significant gender differences in the FCs of the insular subdivisions were revealed: (1) The FCs between the dorsal dysgranular insula (dId) and other brain regions were significantly increased in males using two different techniques (ROI-whole brain and ROI-ROI analyses); (2) Based on the ROI-whole brain analysis, the FC increases in 4 FC-pairs were observed in males, including the left dId - the right median cingulate and paracingulate/ right posterior cingulate gyrus/ right precuneus, the left dId - the right median cingulate and paracingulate, the left dId - the left angular as well as the left dId - the left middle frontal gyrus; (3) According to the ROI-ROI analysis, increased FC between the left dId and the right rostral anterior cingulate cortex was investigated in males.

**Conclusion:**

In summary, the gender differences in the FCs of the insular subdivisions with pain-related brain regions were revealed in the current study, offering neuroimaging evidence for gender differences in pain processing.

**Trial registration:**

ClinicalTrials.gov, NCT02820974. Registered 28 June 2016.

## Background

Pain is one of the most common complaints making people seek medical attention, affecting over one-quarter of the global population, and its incidence increases with aging [1]. Besides aging, gender is another important factor affecting pain procession due to its high prevalence in women [2]. In a large scale study, women report higher pain intensity scores than men under the similar pain conditions [3]. Besides, females are more frequently suffered from pain disorders, such as migraine [4, 5], temporomandibular joint disorder [6], fibromyalgia [7, 8] as well as irritable bowel syndrome (IBS) [9, 10]. These findings suggest that there may be structural or functional gender differences in the brain matrix for pain processing in varied pain disorders [6]. Therefore, a thorough understanding of the central mechanisms underlying this gender differences in healthy people is pivotal for pain research.

Pain experience involves the interaction among sensory, emotional, cognitive, genetic and environmental factors [11] and pain processing is complicated, comprising a variety of brain regions working in concert. Functional magnetic resonance imaging (fMRI) technique has been performed to delineate a set of cortical and subcortical structures-the pain matrix-in pain perception [12, 13]. The classical pain matrix consists of three networks, i.e. the sensory, affective and cognitive networks [14–16]. The sensory network involves the lateral thalamus, primary (SI) and secondary (SII) somatic cortices, the insular cortex as well [17]. Among them, the insular cortex has been suggested to participate in both sensory-discriminative and affective-motivational aspects of pain processing [18]. The stronger pain activations of the somatosensory and the insular cortices in men while medial prefrontal cortex in women under pain challenging have been reported in the previous neuroimaging studies [19–21]. Furthermore, female IBS patients demonstrated significant functional alterations in the insular cortex than male patients [22]. Rather than evaluating evoked responses, it is possible to measure neural activity that is not linked to a specific stimulus or task. The most advanced approach is referred to as “functional connectivity (FC)” [23], which provides insight into how brain regions work together as network to produce pain and how these networks can become strengthened or weakened in pain disorders [24]. Due to the crucial role of insula in pain processing, its FCs with other pain-related brain regions may contribute to interpreting the above-mentioned gender differences. However, the insular cortex is functionally heterogeneous, consisting of multiple distinct subdivisions and the FCs between these subdivisions and other pain processing regions remain largely unknown.

In a recent study, insular cortex is divided into 6 subdivisions and the resting-state FCs between these 6 subdivisions and other 12 pain-related brain regions in both healthy men and women have been reported [25]. However, these subdivisions may not reflect the complexity of insular cortex. Recently, the insular cortex is divided into 12 subdivisions (http://atlas.brainnetome.org/) [26], but it has not been investigated the FCs between these 12 insular subdivisions and more pain-related brain structures, nor is it influenced by gender.

We thus designed the current study to compare the gender differences of FCs between 12 insular subdivisions and other pain-related brain regions by using 2 techniques: first, the gender differences of FCs between the 12 insular subdivisions and the whole brain (region of interest (ROI)-whole brain analysis); second, gender differences of FCs between the 12 insular subdivisions and the 64 selected pain-related brain regions (ROI-ROI analysis).

## Methods

### Participants

Thirty-seven healthy volunteers were recruited from the community participated in the trial, including 26 men (mean age, 49.46 ± 3.75 years) and 11 women (mean age, 52.09 ± 5.65 years). All subjects provided the written informed consent to the study prior to data collection. None participant possessed magnetic resonance imaging (MRI) examination contraindications. Exclusion criteria included neurological diseases or psychiatric disorders, severe internal disorders, concurrence of the cardiovascular system disease, pregnancy or lactation, and alcohol or tobacco dependence.

### Clinical characteristics analysis

The statistical analysis was conducted by using of Statistical Program for Social Sciences (SPSS) 20.0 software. The demographic data of the two groups was compared using a two-sample *t*-test, and *p* < 0.05 was considered as statistical significance.

### Resting-state functional data collection

Resting-state blood oxygenation level dependent (BOLD) images were acquired on GE Discovery MR750 3.0 T MR scanner with an eight-channel phased-array head coil at the Department of Radiology, Tangdu Hospital of the Fourth Military Medical University. Prior to scanning, the head of each subject was stabilized in order to minimize head motion. Moreover, the head position was monitored during the whole scanning. For each subject, structural T1 weighted imaging (T1WI) and T2 fluid attenuated inversion recovery (FLAIR) sequences were firstly employed to exclude apparent brain lesions. Resting-state BOLD images were collected utilizing an echo planar imaging (EPI) sequence. Through the scanning process, participants were placed in the supine position and informed to remain as motionless as possible, keep awake, relax their minds and think of nothing in particular. The EPI sequence scanning lasted for 6 min and 10 s. The scanning parameters were as the following:

T1WI:flip angle (FA) = 111°, echo time (TE) = 24 ms, number of echoes = 1, repetition time (TR) = 1750 ms, inversion time (TI) = 780 ms, receiver bandwidth = 41.67, field of view (FOV) = 24 mm^2^, slice thickness = 5 mm, slice spacing = 1.5, number of slices = 20;

T2 FLAIR: TE = 145 ms, number of echoes = 1, TR = 8400 ms, TI = 2100 ms, receiver bandwidth = 83.33, FOV = 24 mm^2^, slice thickness = 5 mm, slice spacing = 1.5, number of slices = 18;

BOLD: FA = 90°, TE = 30 ms, number of echoes = 1, TR = 2000 ms, number of shots = 1, FOV = 22 mm^2^, slice thickness = 3 mm, slice spacing = 1.0, number of slices = 36, time points = 185.

### Brain region masks

#### Masks for insular subdivisions

According to the published Brainnetome Atlas (http://atlas.brainnetome.org/download.html), the insular cortex is divided into 12 subdivisions, including the hypergranular (G), ventral agranular (vIa), dorsal agranular (dIa), ventral dysgranular and granular (vId/vIg), dorsal granular (dIg), dorsal dysgranular (dId) in left and right insular cortices [27]. The detailed information of the 12 insular subdivisions was presented in Table 1.

**Table 1:** MNI coordinates of the 12 insular subdivisions

Insular subdivision	Abbreviation	Left and right hemisphere	Label	MNI coordinate		
				X	Y	Z
Hypergranular insula	G	L	G_L	−36	−20	10
Hypergranular insula	G	R	G_R	37	−18	8
Ventral agranular insula	vIa	L	vIa_L	−32	14	−13
Ventral agranular insula	vIa	R	vIa_R	33	14	−13
Dorsal agranular insula	dIa	L	dIa_L	−34	18	1
Dorsal agranular insula	dIa	R	dIa_R	36	18	1
Ventral dysgranular and granular insula	vId/vIg	L	vId/vIg_L	−38	−4	−9
Ventral dysgranular and granular insula	vId/vIg	R	vId/vIg_R	39	−2	−9
Dorsal granular insula	dIg	L	dIg_L	−38	−8	8
Dorsal granular insula	dIg	R	dIg_R	39	−7	8
Dorsal dysgranular insula	dId	L	dId_L	−38	5	5
Dorsal dysgranular insula	dId	R	dId_R	38	5	5

*L* left, *R* right, *MNI* Montreal Neurological Institute

#### Masks for selected pain-related brain regions

Based on the neuroanatomical knowledge of brain as well as extensive literature reviewing, 64 pain-related brain regions were selected in the current fMRI study. The detailed information of these pain-related brain regions, including the location, label and Montreal Neurological Institute (MNI) coordinate, was shown in Table 2. Masks for most pain-related regions were selected from the Brainnetome Atlas, including ventral dorsolateral prefrontal cortex (vDLPFC), the opercular pars triangularis (oPT) and ventral pars triangularis (vPT), primary motor cortex (PMC), postcentral somatosensory association cortex (pSAC), the caudal supramarginal gyrus (cSG) and rostroventral supramarginal gyrus (rvSG), SI, the rostroventral ventral anterior cingulate cortex (rvVACC), caudal ventral anterior cingulate cortex (cvACC), pregenual dorsal anterior cingulate cortex (pdACC) and subgenual dorsal anterior cingulate cortex (sdACC), the dorsolateral putamen (dlPu), the medial prefrontal thalamus (mPFtha), premotor thalamus (mPMtha), posterior parietal thalamus (PPtha), caudal temporal thalamus (cTtha), and lateral prefrontal thalamus (lPFtha), the medial amygdala (mAmyg) and lateral amygdala (lAmyg), the rostral hippocampus (rHipp) and caudal hippocampus (cHipp). Besides, the mask for SII was chosen from the Juelich Histological Atlas, which was distributed with FMRIB Software Library (FSL) tool. In addition, the specific MNI coordinates of the rest pain-related regions were referred to the published studies, including rostral anterior cingulate cortex (rACC), ventrolateral prefrontal cortex (VLPFC), posterior midcingulate cortex (pMCC), orbitofrontal cortex (OFC) as well as the ventrolateral periaqueductal gray (vlPAG), lateral periaqueductal gray (lPAG), and dorsolateral periaqueductal gray (dlPAG) [28].

**Table 2:** MNI coordinates of the 64 selected pain-related brain regions

Location	Brain region	Abbreviation	Left and right hemisphere	Label	MNI coordinate		
					X	Y	Z
Middle frontal gyrus	ventral dorsolateral prefrontal cortex	vDLPFC	L	vDLPFC_L	−41	41	16
Middle frontal gyrus	ventral dorsolateral prefrontal cortex	vDLPFC	R	vDLPFC_R	42	44	14
Middle frontal gyrus	ventrolateral prefrontal cortex	VLPFC	L	VLPFC_L	−48	20	−8
Middle frontal gyrus	ventrolateral prefrontal cortex	VLPFC	R	VLPFC_R	48	20	−8
Inferior frontal gyrus	opercular pars triangularis	oPT	L	oPT_L	−39	23	4
Inferior frontal gyrus	opercular pars triangularis	oPT	R	oPT_R	42	22	3
Inferior frontal gyrus	ventral pars triangularis	vPT	L	vPT_L	−52	13	6
Inferior frontal gyrus	ventral pars triangularis	vPT	R	vPT_R	54	14	11
Frontal gyrus	orbitofrontal cortex	OFC	L	OFC_L	−24	34	−12
Frontal gyrus	orbitofrontal cortex	OFC	R	OFC_R	24	34	−12
Precentral gyrus	primary motor cortex	PMC	L	PMC_L	−52	0	8
Precentral gyrus	primary motor cortex	PMC	R	PMC_R	54	4	9
Superior parietal lobe	postcentral somatosensory association cortex	pSAC	L	pSAC_L	−22	−47	65
Superior parietal lobe	postcentral somatosensory association cortex	pSAC	R	pSAC_R	23	−43	67
Inferior parietal lobe	caudal supramarginal gyrus	cSG	L	cSG_L	−56	−49	38
Inferior parietal lobe	caudal supramarginal gyrus	cSG	R	cSG_R	57	−44	38
Inferior parietal lobe	rostroventral supramarginal gyrus	rvSG	L	rvSG_L	−53	−31	23
Inferior parietal lobe	rostroventral supramarginal gyrus	rvSG	R	rvSG_R	55	−26	26
Postcentral gyrus	primary somatosensory cortex (tongue and larvnx region)	SI	L	SI_L	−56	−14	16
Postcentral gyrus	primary somatosensory cortex (tongue and larvnx region)	SI	R	SI_R	56	−10	15
Postcentral gyrus	primary somatosensory cortex (trunk region)	SI	L	SI_L	−21	−35	68
Postcentral gyrus	primary somatosensory cortex (trunk region)	SI	R	SI_R	20	−33	69
Postcentral gyrus	secondary somatosensory cortex	SII	L	SII_L	−52	−26	22
Postcentral gyrus	secondary somatosensory cortex	SII	R	SII_R	56	−22	24
Cingulate gyrus	rostroventral ventral anterior cingulate cortex	rvVACC	L	rvVACC_L	−3	8	25
Cingulate gyrus	rostroventral ventral anterior cingulate cortex	rvVACC	R	rvVACC_R	5	22	12
Cingulate gyrus	caudal ventral anterior cingulate cortex	cvACC	L	cvACC_L	−5	7	37
Cingulate gyrus	caudal ventral anterior cingulate cortex	cvACC	R	cvACC_R	4	6	38
Cingulate gyrus	pregenual dorsal anterior cingulate cortex	pdACC	L	pdACC_L	−6	34	21
Cingulate gyrus	pregenual dorsal anterior cingulate cortex	pdACC	R	pdACC_R	5	28	27
Cingulate gyrus	subgenual dorsal anterior cingulate cortex	sdACC	L	sdACC_L	−4	39	−2
Cingulate gyrus	subgenual dorsal anterior cingulate cortex	sdACC	R	sdACC_R	5	41	6
Cingulate gyrus	rostral anterior cingulate cortex	rACC	L	rACC_L	−7	27	29
Cingulate gyrus	rostral anterior cingulate cortex	rACC	R	rACC_R	7	27	29
Cingulate gyrus	caudal ventral posterior cingulate cortex	cvPCC	L	cvPCC_L	−7	−23	41
Cingulate gyrus	caudal ventral posterior cingulate cortex	cvPCC	R	cvPCC_R	6	−20	40
Cingulate gyrus	posterior midcingulate cortex	pMCC	L	pMCC_L	−3	−21	51
Cingulate gyrus	posterior midcingulate cortex	pMCC	R	pMCC_R	3	−21	51
Basal ganglia	dorsolateral putamen	dlPu	L	dlPu_L	−28	−5	2
Basal ganglia	dorsolateral putamen	dlPu	R	dlPu_R	29	−3	1
Periaqueductal gray	ventrolateral periaqueductal gray	vlPAG	L	vlPAG_L	−3	−32	−12
Periaqueductal gray	ventrolateral periaqueductal gray	vlPAG	R	vlPAG_R	3	−32	−12
Periaqueductal gray	lateral periaqueductal gray	lPAG	L	lPAG_L	−4	−31	−8
Periaqueductal gray	lateral periaqueductal gray	lPAG	R	lPAG_R	4	−31	−8
Periaqueductal gray	dorsolateral periaqueductal gray	dlPAG	L	dlPAG_L	−2	−32	−5
Periaqueductal gray	dorsolateral periaqueductal gray	dlPAG	R	dlPAG_R	2	−32	−5
Thalamus	medial prefrontal thalamus	mPFtha	L	mPFtha_L	−7	−12	5
Thalamus	medial prefrontal thalamus	mPFtha	R	mPFtha_R	7	−11	6
Thalamus	premotor thalamus	mPMtha	L	mPMtha_L	−18	−13	3
Thalamus	premotor thalamus	mPMtha	R	mPMtha_R	12	−14	1
Thalamus	posterior parietal thalamus	PPtha	L	PPtha_L	16	−24	6
Thalamus	posterior parietal thalamus	PPtha	R	PPtha_R	15	−25	6
Thalamus	caudal temporal thalamus	cTtha	L	cTtha_L	−12	−22	13
Thalamus	caudal temporal thalamus	cTtha	R	cTtha_R	10	−14	14
Thalamus	lateral prefrontal thalamus	lPFtha	L	lPFtha_L	−11	−14	2
Thalamus	lateral prefrontal thalamus	lPFtha	R	lPFtha_R	13	−16	7
Amygdala	medial amygdala	mAmyg	L	mAmyg_L	−19	−2	−20
Amygdala	medial amygdala	mAmyg	R	mAmyg_R	19	−2	−19
Amygdala	lateral amygdala	lAmyg	L	lAmyg_L	−27	−4	− 20
Amygdala	lateral amygdala	lAmyg	R	lAmyg_R	28	−3	−20
Hippocampus	rostral hippocampus	rHipp	L	rHipp_L	−22	−14	−19
Hippocampus	rostral hippocampus	rHipp	R	rHipp_R	22	−12	−20
Hippocampus	caudal hippocampus	cHipp	L	cHipp_L	−28	−30	−10
Hippocampus	caudal hippocampus	cHipp	R	cHipp_R	29	−27	−10

*L* left, *R* right, *MNI* Montreal Neurological Institute

### Processing of resting-state fMRI data

#### Data preprocessing

The preprocessing of the BOLD images was performed by using the Data Processing Assistant for Resting-State fMRI (DPARSF, Yan and Zang 2010, http://rfmri.org/DPARSF), which is based on Statistical Parametric Mapping (SPM8, http://www.fil.ion.ucl.ac.uk/spm) and the toolbox for Data Processing & Analysis of Brain Imaging (DPABI, Yan et al. 2016, http://rfmri.org/DPABI). Briefly, the first 10 volumes of each subject were discarded in order to reach the signal equilibration and allow the subjects to adapt to the scanning environment. The remained scans were corrected for acquisition time differences between different slices and next realigned to the middle time point to correct for head motion. Then the head motion parameters of the subject were obtained and assessed with a maximum rotation less than 1° or a maximum displacement less than 1 mm. Nuisance regression was applied on white matter and cerebrospinal fluid, separately. Then the motion-corrected BOLD images were spatially normalized by using EPI templates and resampled with a voxel size of 3 × 3 × 3 mm^3^. After spatial normalization, the images were spatially smoothed with a Gaussian kernel of 4 mm full-width at half maximum (FWHM) to reduce spatial noise. In the end, a band-pass filter (0.01 Hz < f < 0.08 Hz) was utilized to remove the effects of low frequency physiological drift and high frequency noise. After performing these steps, a 4-dimensional residual time series dataset was set up for each subject in the standard MNI space.

#### FCs analysis between the 12 insular subdivisions and the whole brain

The ROI-whole brain analysis was conducted between the 12 insular subdivisions (a total of 12 ROIs) and the whole brain. The mean time series for each ROI were calculated and then correlated with the time courses of all other voxels in the brain for each subject. Pearson correlation coefficients were converted to normally distributed scores by use of the Fisher’s r-to-z transformation [23]. Group-level analysis for the general linear model was performed applying two sample *t*-test between the z-scores of the male and female groups. The reported results of the ROI-whole brain correlation analysis were carried out performing an uncorrected peak level of *p* < 0.001 to correct for false positive rates and a false discovery rate (FDR) correction by using SPM8 at cluster level for multiple comparisons with threshold of *p* < 0.05 [29, 30].

#### FCs analysis between the 12 insular subdivisions and the 64 selected pain-related regions

Next, 64 brain regions that were recognized to be associated with pain were selected to calculate the ROI-ROI level FCs with the 12 sub-insular divisions (a total of 12 ROIs). Similar to the above-mentioned ROI-whole brain level analysis, Fisher’s r-to-z transformation was conducted to increase the normality of the fMRI data. Based on the 12 × 76 z-FC matrix, two-sample *t*-test was performed between the male group and the female group. FDR correction with threshold of *p* < 0.05 for multiple comparison was then employed using MATLAB 2012b and to identify the final FC pairs with significant difference attributed to the gender factor.

## Results

### Demographic data

The demographic data of the two groups were shown in Table 3. No significant differences were revealed between male and female groups in age, hand dominance as well as educational level.

**Table 3:** Demographic characteristics

	Male group (*n* = 26)	Female group (*n* = 11)	*P*
Age (years)	49.46 ± 3.75	52.09 ± 5.65	0.104
Hand dominance (L/R)	2/24	1/10	1.000
Education (years)	13.54 ± 3.27	12.18 ± 3.22	0.259

Continuous data were expressed as mean ± standard deviation (SD)

*L* left, *R* right

### Gender differences in ROI-whole brain level FCs

**Fig. 1:**
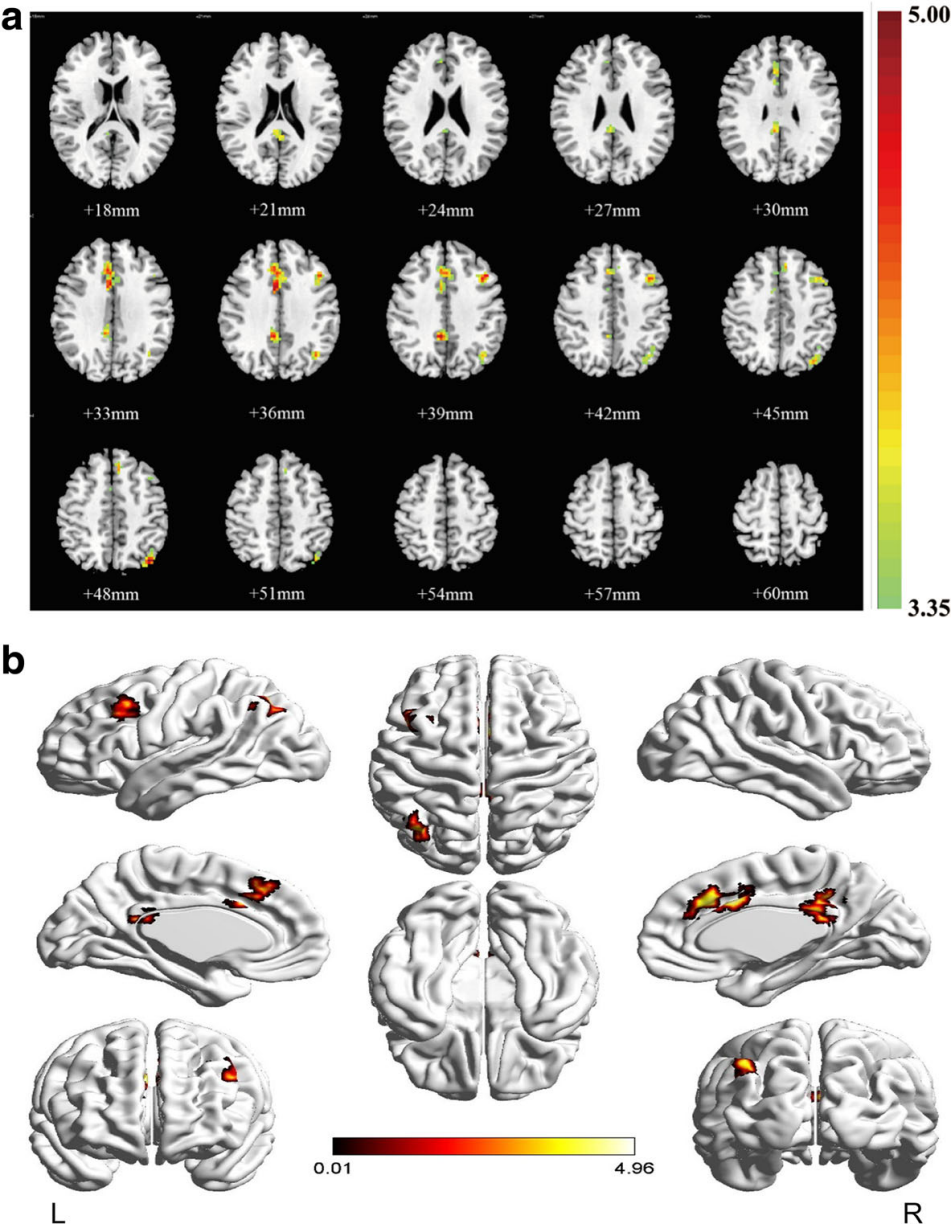
ROI-whole brain analysis of FCs between the 12 insular subdivisions and the whole brain (visualization of the clusters on the brain surface). The warm color in the statistical differences map indicated the increased FCs between the dId_L and the whole brain in males. dId, dorsal dysgranular insula; L, left; R, right; FCs, functional connectivities; ROI, region of interest

FCs between the 12 insular subdivisions and whole brain in male and female groups were first investigated. Male subjects showed significantly increased FCs between the left dId and other four brain regions, comprising the voxels containing the right median cingulate and paracingulate/ right posterior cingulate gyrus/ right precuneus, the right median cingulate and paracingulate, the left angular, and the left middle frontal gyrus (Table 4 and Fig. 1a). The surface visualization of these significantly increased clusters in males was presented in Fig. 1b.

**Table 4:** Significant FCs between the insular subdivision and the whole brain in males

Insular subdivision	Cluster index	Brain region	Cluster size	MNI coordinate			Peak T-value	FCs increased/ decreased
				X	Y	Z		
dld_L	1	Cingulum_Mid/Cingulum_Post/Precuneus_R	73	6	−45	39	4.82	Increased
	2	Cingulum_Mid_R	144	6	9	36	4.76	Increased
	3	Angular_L	71	−39	−72	48	4.96	Increased
	4	Frontal_Mid_L	56	−42	21	39	4.53	Increased

The threshold was set at uncorrected peak level of *p* < 0.001, FDR correction with threshold of *p* < 0.05, cluster size ≥50 voxels. T-values of significantly activated peak-voxels referred to MNI coordinates. Brain region labeling was performed using the AAL atlas. T statistics and MNI coordinates were reported for the peak voxel within each cluster. AAL, Automatic Anatomic Labeling; FCs, functional connectivities; L, left; R, right; MNI, Montreal Neurological Institute; dld, dorsal dysgranular insula; Cingulum_Mid, median cingulate and paracingulate gyrus; Cingulum_Post, posterior cingulate gyrus; Frontal_Mid, middle frontal gyrus

### Gender differences in ROI-ROI level FCs

**Fig. 2:**
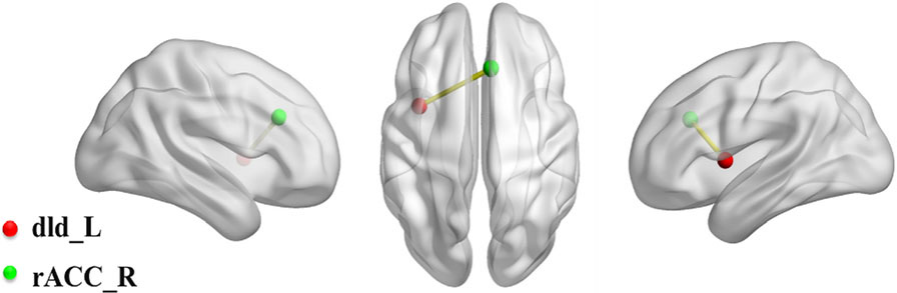
ROI-ROI analysis of FCs between the 12 insular subdivisions and 64 selected pain-related brain regions. The red ball represented the dId_L, the green ball represented the rACC_R, and the yellow rod represented the statistically increased FC in males. L, left; R, right; dId, dorsal dysgranular insula; rACC, rostral anterior cingulate cortex; ROI, region of interest; FCs, functional connectivities

FCs between the insular subdivisions and selected pain-related brain regions in male and female groups were compared using ROI-ROI level analysis. In line with the above ROI-whole brain analysis, the significant gender differences of FCs were also investigated between the 12 insular subdivisions and the 64 selected pain-related brain regions. Only the FC between the left dId and the right rACC in the total 12 × 76 z-FC matrix was increased in the male subjects. The gender differences of 1 FC-pair with significance between the insular subdivisions and pain-related brain regions were shown in Fig. 2.

**Fig. 3:**
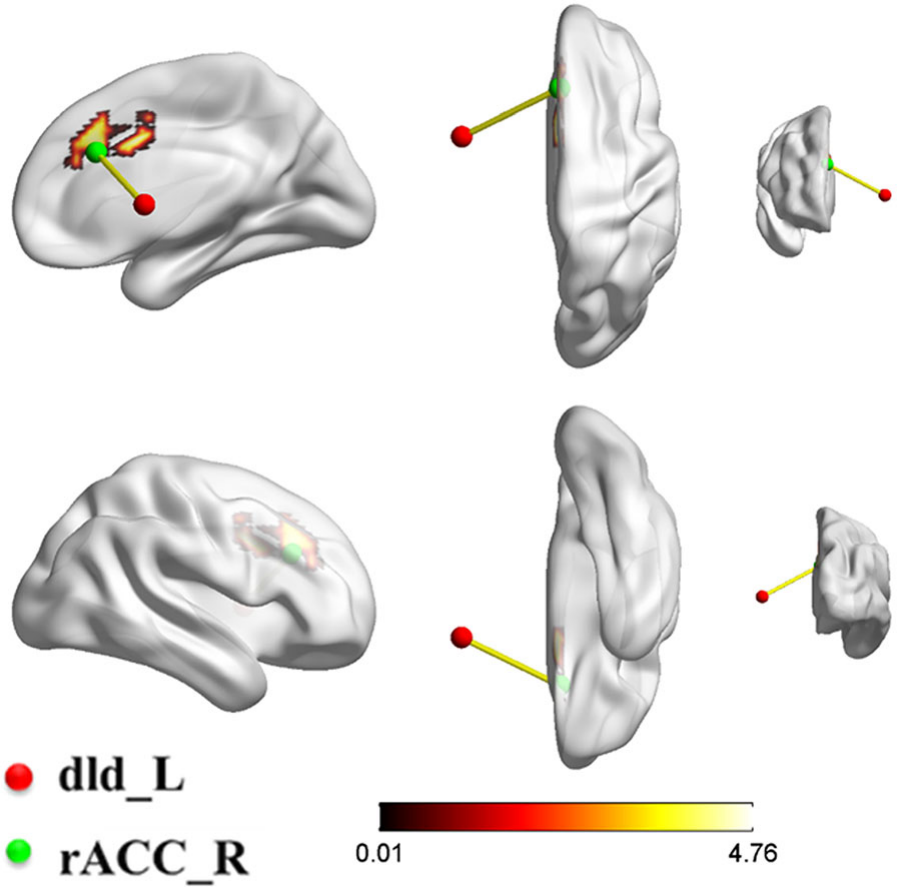
Merging of ROI-whole brain and ROI-ROI analyses. The warm color in the statistical differences map exhibited the increased FCs between the dId_L and the whole brain in males (ROI-whole brain analysis). The red ball represented the dId_L, the green ball represented the rACC_R, and the yellow rod represented the statistically increased FC between the dId_L and the rACC_R in males **(**ROI-ROI analysis**)**. L, left; R, right; dId, dorsal dysgranular insula; rACC, rostral anterior cingulate cortex; ROI, region of interest; FCs, functional connectivities

After merging the results of ROI-whole brain and ROI-ROI analyses, the dId in the left insular cortex was the primary insular subdivision that showed significantly increased FCs with the brain structures predominantly located in the right cingulate cortex in male subjects, shown in Fig. 3.

## Discussion

In the current study, two analytical approaches were performed to explore the gender differences of FCs in insular subdivisions. Both ROI-whole brain and ROI-ROI analyses suggested that the FCs between left dId and right cingulate cortex were significantly increased in males. Our findings suggested that the increased FCs between left dId - right cingulate cortex in males played inhibiting role in the transmission and modulation of pain signals, thus decreased the pain intensity and incidence in male population.

According to the cytoarchitecture of the insular cortex, the dId locates in the posterior area of insula and participates in the sensory components of pain perception [27, 31]. In migraine patients, the dId was negatively connected to the median cingulate and paracingulate [25]. Female migraine patients also exhibited significantly decreased FCs between the posterior insula and the posterior cingulate cortex [5]. In addition to the above-mentioned gender differences in the clinical experience of migraine pain, healthy males in the current study showed statistically increased FCs between the insular subdivision and the cingulate cortex. Taking together the previous studies and our current one, the FCs between the insular and cingulate cortices were different between males and females, suggesting the functional distinctions in the brain matrix for pain processing among male and female populations.

Previous fMRI studies on gender differences in healthy subjects suggested that the male insular cortex is more activated under similar pain stimuli [19], however, without details of the activated subdivisions. Our study offered evidence that the male dorsal dysgranular area of the posterior insula was more spontaneously activated than females. Since the posterior insula primarily plays an important role in the sensory network of pain [32, 33], our data suggested the potential involvement of dorsal dysgranular area of the posterior insula in the different pain processing in males and females under pain disorders. Reduced activity in the posterior insula may contribute to increased pain thresholds [34], suggesting its role in inhibiting pain response. However, the detailed underlying mechanisms need to be further investigated.

To our knowledge, information exchange between the insular and cingulate cortices has never been directly revealed. However, tracing studies in the monkey demonstrated that the posterior insula was connected to Brodmann areas 23 and 24 of the cingulate cortex [35], supporting our current findings. Based on the non-human primate functional neuroimaging studies, a co-activation of the insular and cingulate cortices was revealed in varied tasks [32, 36]. Furthermore, the posterior insular and the cingulate cortices were activated simultaneously in fibromyalgia patients [37]. Therefore, the distinct anatomical connections identified in the monkey combined with the evidence from functional imaging studies showing co-activation within specific areas of the insula and cingulate cortex, indicating multiple information processing pathways between the two brain structures [36]. Interestingly, the co-activation of the insular subdivision and the cingulate cortex also showed the gender differences in the current study. The FCs between the dId and cingulate cortex were significantly increased in male subjects. The FCs between insular subdivisions and other pain-related brain regions were also investigated in a recent study [25]. Despite the similar study design, there were apparent differences between their findings and ours. First, Wiech explored FCs between the insular subdivisions and other pain-related regions using the ROI-ROI method [25]. While we aimed to investigate that how the FCs between the insular subdivisions and other pain-relevant regions were influenced by gender difference (ROI-ROI analysis). In addition, we also identified the gender differences in FCs between the insular subdivisions and the whole brain (ROI-whole brain analysis). Second, the insular cortex was divided into 6 subdivisions in the previous study, while 12 subdivisions were utilized in the current study. Third, 12 pain-related brain regions were selected in Wiech’s study, while 64 pain-related regions were selected in the present study based on an existing neuroanatomical knowledge and extensive literature review. Wiech revealed strongest FCs between the posterior insula and SII in 36 healthy adults (21 men and 15 women). Our study offered further information that the dId of the posterior insula is a major hub to cingulate cortex, performing pain inhibiting role.

There was limitation for our study. No pain-related psychological questionnaires were collected in the current study. Based on the existing evidence, healthy subjects also possess the pain-relevant psychological characteristics, such as pain vigilance and pain awareness [25]. The correlation analysis might reveal the potential relation between the FCs and the psychological traits of pain.

## Conclusion

In conclusion, the present study identified the gender-relevant alterations in FCs of the insular subdivisions with other pain-related brain regions using two different methods. Specifically, men seem to have more access to a dId-mediated recruitment of the pain inhibition system than women. Given this, it is crucial for future study to take gender into account when probing the basic mechanisms of pain processing.

## Abbreviations

AAL: Automatic anatomic labeling
BOLD: Blood oxygenation level dependent
DPABI: Data processing & analysis of brain imaging
DPARSF: Data processing assistant for resting-state fMRI
EPI: Echo planar imaging
FA: Flip angle
FC: Functional connectivity
FLAIR: Fluid attenuated inversion recovery
fMRI: Functional magnetic resonance imaging
FOV: Field of view
FSL: FMRIB software library
FWHM: Full-width at half maximum
IBS: IRRITABLE bowel syndrome
L: Left
MNI: Montreal neurological Institute
MRI: Magnetic resonance imaging
R: Right
ROI: Regions of interest
SD: Standard deviation
SI: Primary somatic cortices
SII: Secondary somatic cortices
SPM: Statistical parametric mapping
SPSS: Statistical program for social sciences
T1WI: T1 weighted imaging
TE: Echo time
TI: Inversion time
TR: Repetition time
VLPFC: Ventrolateral prefrontal cortex
